# Lifetime utilization of mammography among Maltese women: *a cross-sectional survey*

**DOI:** 10.1186/s12889-018-5093-6

**Published:** 2018-01-25

**Authors:** Danika Marmarà, Vincent Marmarà, Gill Hubbard

**Affiliations:** 1 Faculty of Health Sciences, University of Stirling, Room E9, Pathfoot, Stirling, FK9 4LA Scotland; 2 Ministry for Health, Cancer Care Pathways Directorate, Sir Anthony Mamo Oncology Centre, Level -1, Dun Karm Psaila Street, Msida, MSD 2090 Malta; 3 Department of Management, University of Malta, Msida, Malta

**Keywords:** Breast cancer, Mammography, Attendance, Non-attendance, Health beliefs, Illness perceptions

## Abstract

**Background:**

The knowledge of Maltese women not attending the Maltese Breast Screening Programme (MBSP) for mammography screening is scarce. Previous research has identified two distinct groups of non-attendees: those who do not attend because a mammogram was taken elsewhere and those who never attended for mammography anywhere. It is however unknown which determinants are predictive of lifetime attendance ‘anywhere’ and ‘real’ non-attendance. The present study examines the relationship between ever-using (Lifetime attendees) or never using mammography (Lifetime non-attendees) and psychosocial - as well as sociodemographic factors, with the aim to identify predictors that can inform practice.

**Methods:**

Women’s characteristics, knowledge, health beliefs and illness perceptions were compared, based on prior data of 404 women, aged 50–60 years at the time of their first MBSP invitation. The main variable of interest described women’s attendance to mammography (LIFETIME ATTENDEES) and no mammography (LIFETIME NON-ATTENDEES). Data were analyzed using descriptive statistics, chi-square tests, Mann Whitney test, Independent Samples t-test, Shapiro Wilk test and logistic regression.

**Results:**

During their lifetime, 86.1% of Maltese women (*n* = 348) were attendees, while 13.9% (*n* = 56) were non-attendees. Non-attendees were more likely to be women with a lower family income (χ2 = 13.1, *p* = 0.011), widowers (χ2 = 9.0, *p* = 0.030), non-drivers (χ2 = 7.7, *p* = 0.006), without a breast condition (χ2 = 14.2, *p* <  0.001), who had no relatives or close friends with cancer (χ2 = 8.3, *p* = 0.016), and who were less encouraged by a physician (χ2 = 4.9, *p* = 0.027), unsure of the screening frequency (χ2 = 28.5, *p* <  0.001), more anxious (*p* = 0.040) and fearful (*p* = 0.039). Perceived benefits, barriers, cues to action, self-efficacy and emotional representations were the most significant variables to describe the differences between lifetime attendees and non-attendees. Perceived barriers and cues to action were the strongest predictors for lifetime non-attendance (*p* <  0.05 respectively).

**Conclusions:**

The health beliefs of women who have never attended for mammography during their lifetime should be targeted, particularly perceived barriers and cues to action. Further research should focus on understanding knowledge gaps, attitudinal barriers and emotional factors among ‘real’ non-attendees who require a more targeted approach.

## Background

Breast cancer (BC) is the most common type of cancer in women worldwide [1]. In Malta, it has topped the list of female cancers and has accounted for an average incidence of over 280 women over the last 12 years [2]. Early detection of BC renders the possibility of efficient treatment [3] which would more likely include breast conservation without chemotherapy [4]. Regular use of mammography screening at short enough intervals is a cost-effective way [5] to detect tumours early enough in order to improve prognosis, reducing mortality and thereby impacting on survival [4, 6, 7].

Across the globe, lifetime utilization and regular re-utilization of mammography has been increasing steadily across the years [3, 7–9]. Despite the known benefits of breast screening (BS) by mammography [10–12], also referred to as mammography screening (MS), various countries have still not reached the recommended acceptable (> 70%) or desirable (> 75%) EU benchmarks, according to the European Guidelines [13]. Lower utilization rates may be associated with three main factors: (a) *logistical determinants* such as the availability and accessibility of a screening center, test affordability, time from work or travelling time [3, 14, 15], (b) *psychosocial factors* such as values, expectations and beliefs which affect the way women transform knowledge regarding mammography into actual behaviour [16], and (c) socio-demographic determinants which impact on the way structural and psychosocial factors predict mammography use [17, 18]. However, most of the literature does not take into account the context of mammography provision, such as countries with dual health systems (organized and private screening).

Although general barriers to screening by mammography in Malta have been identified in our earlier study [19], our findings showed that our screening cohort consisted of attendees and non-attendees to the Maltese Breast Screening programme (MBSP); however, we recognised that the MBSP non-attendees consisted of a heterogeneous group of women with diverse reasons for non-attendance. Hence, screening non-attendees were not a single group of non-compliant Maltese women, but consisted clearly of two distinct subgroups:

(i) Women, who had obtained a mammogram outside the MBSP, possibly as a self-initiated action or routine check-up [15] or as part of private breast awareness campaigns, which may have been based on their recognition of susceptibility to BC and high self-efficacy in preventing BC [20], and
(ii) ‘Real’ non-attendees i.e. women who have never attended anywhere for mammography during their lifetime.

Considering the fact that the Maltese National Health System (NHS) comprises both the public and private sectors, and that a national breast screening programme was introduced at the end of 2009 for women aged 50–60 years at the time [13], some women chose to go privately for a mammogram before the year 2009 and still do so to date rather than taking up the invitation to be screened at the MBSP. However, it is the diversity of ‘real’ non-attendees [15] that needs to be better understood in order to develop culturally sensitive interventions.

Nothing is yet known to date about those who never attend for mammography throughout their lifetime in Malta. Hence, this study was carried out to provide an understanding of the determinants of lifetime mammography screening behaviour among Maltese women who attend ‘anywhere’ and those who have ‘never’ attended for mammography. This paper is “part two” of a larger study that was conducted on breast screening uptake in Malta carried out through a national cross-sectional survey and hence, this paper is a continuation of that previous article. In this paper, data from that 2015 Maltese national survey were used to assess the relationships of lifetime mammography utilization (attendance ‘anywhere’) and ‘real’ non-attendance with socio-demographics, health status, knowledge, health beliefs and illness perception variables, based on the Health Belief Model (HBM) and Common-Sense Model (CSM). Both HBM and CSM have been used as theoretical frameworks to predict the uptake of mammography screening [21–24]. The CSM has been used to consider the cognitive and emotional representations of an illness [23] which are often omitted in the use of HBM. On the other hand, the CSM does not describe the perceived barriers and benefits to the performance of health-related behaviours [21] such as mammography use, and excludes the role of significant others such as family, friends and healthcare providers [25]. By contrast, the HBM addresses all of these, incorporating the components of perceived benefits, perceived barriers and cues to action. Following the simultaneous use of both models which were found to improve the prediction of non-attendance to the MBSP in our earlier study [19], both models were again utilised to integrate the beliefs about the illness (CSM) [26] and the individual’s beliefs on the recommended behaviour [HBM] [21] in this study on lifetime mammography utilization.

Guided by the guidelines ‘Strengthening the Reporting of Observational Studies in Epidemiology’ (STROBE) [27] [see Additional file 1], we built on the findings of our prior study [28] which suggest that health beliefs and illness perceptions vary between women who accept or refuse a BS invitation to the organized programme. The immediate aim of our study was to gain an understanding of the determinants of lifetime mammography use among women who attend for mammography ‘anywhere’ and those who never attend for mammography during their lifetime (‘real’ non-attendance).

### Objectives

In reaching our aims, this analysis has targeted the following objectives:

1. To determine the socio-demographics, health status, knowledge, health beliefs and illness perceptions of women who attend or do not attend for mammography screening during their lifetime;
2. To examine the most significant predictors of lifetime mammography utilization and its non-use.

## Methods

### Design and setting

Since this study was part of a 2015 national retrospective study, the full details of the methods are described elsewhere [19]. The MBSP was set up to serve as the only centre in Malta to offer national screening as part of an organized programme. As is the current practice at the MBSP, two views (medio-lateral and cranio-caudal) are carried out by trained radiographers (mammographers) and the mammograms are reported by trained breast radiologists. Adjunct ultrasound is carried out at a subsequent (recall) appointment when deemed necessary, for cases such as dense breasts or for further evaluation of suspected mammographic abnormalities.

A stratified random sample was ascertained from women aged 50–60 at the time of their first invitation at the MBSP who were registered on the MBSP database and who had no personal history of BC. The original study recruited a sample size of 404 women (i.e. 243 attendees and 161 non-attendees) in order to achieve a 95% confidence level and 5% confidence interval, which the present study used.

For those invited to the MBSP, attendance or non-attendance was verified through screening records but further mammography performed in private practices was self-reported. Participants were assured that their participation was voluntary and that they could withdraw from the study at any time without the need to give a reason. Information was provided to the women on how the researcher would protect their anonymity and confidentiality through coding. Prior to the commencement of the survey, participants were informed that the study was aimed at improving the understanding of women’s beliefs, attitudes and perceptions on and concerns about BS and BC. Moreover, they were notified that the study had been granted ethical approval by the School Research Ethics Committee at the University of Stirling (SREC14/15-Paper No.18v4) and by the Maltese Health Ethics Committee (HEC 02/2015). As approved by the ethics committees and following standard practice when conducting surveys by telephone [29–31], a research assistant was responsible for participant recruitment, which was carried out manually over the phone (through “yes” or “no” response options), using paper format to record verbal informed consent. Following the latter method, an appointment was scheduled by the research assistant to match its suitability for each of the participants and the primary investigator (DM). The survey was completed in a median of 25 min (range, 15–45 min) and was carried out in one telephone call.

### Survey development

The survey questionnaire consisted of standardized socio-demographic and health status questions as well as validated scales (CHBMS-MS and IPQ-R) [32, 33]. All measures were translated from English to Maltese using a back-translation procedure. A pre-test (*n* = 15) of the 121-item tool (entitled the Maltese Breast Screening Questionnaire – MBSQ) confirmed the comprehensibility, accuracy and feasibility of the questionnaire and to ensure understanding of scale items in both Maltese and English. The methods used have been published elsewhere [19, 34].

The survey questionnaire is composed of four sections, as follows:

1. Socio-demographic factors and health status were measured through 11 subscales (20 items),
2. Lifetime mammography practices and knowledge of mammography frequency were measured through 4 subscales (17 items),
3. Health beliefs were measured through 5 subscales (36 items),
4. Illness perceptions were measured through 7 subscales (48 items).

Response options were “yes”, “no” or a series of tick boxes for socio-demographic factors and health status variables. Open questions were designed to encourage a more detailed and meaningful answer using the participant’s own knowledge and/or feelings. Most of the response options for lifetime mammography practices and knowledge on mammography time intervals were mostly designed to elicit “yes”, “no” or “unsure” answers, whereas closed questions were possible through a series of tick boxes. All items for health beliefs and illness perceptions had 5 response options (1 = ‘strongly disagree’ to 5 = ‘strongly agree’).

### Classification of variables

Women were asked if they ever had a mammogram in their lifetime with a yes/no response. Women were categorized as LIFETIME ATTENDEES if they had *ever* had a mammogram in their lifetime or LIFETIME NON-ATTENDEES if they had *never* attended for a mammogram during their lifetime. Socio-demographic and health status variables (some of which were confirmed from women’s health records from the screening database), as well as knowledge of screening frequency, health beliefs and illness perception variables were collected from the survey administered retrospectively from the time of the first screening invitation at the MBSP.

### Statistical analysis

The chi-square test was used for comparison of proportions between two categorical variables. The Shapiro Wilk test was applied on the 14 constructs in order to determine whether these variables are normally distributed. It was found that only the variable Causes of BC was normally distributed. Hence, parametric tests were used for this latter construct. All the other 13 constructs were found to be not normally distributed (*p*-value < 0.001) and hence, non-parametric tests were used for all the 13 constructs. When comparing two independent samples, the Independent Samples t-test was used for normally distributed data (parametric test) and Mann-Whitney test was used for the non-normal distributed dataset (non-parametric test). Similarly, for analysis including two of more independent samples, ANOVA was used for normally distributed data and Kruskal-Wallis test was used for the non-normal distributed datasets. Different variables and constructs were incorporated into six logistic regression models and the ‘backward-elimination’ method was applied to each model to identify the significant predictors of lifetime mammography use. The results of the regression are reported with 95% confidence intervals, Beta (unstandardized) coefficients, Standard Errors (SE), Walds, Odds Ratios (OR) and *p*-values. All tests were analysed with an α = 0.05 level of significance; hence, any statistical test obtaining a p-value of < 0.05 was considered as statistically significant. Missing data was minimal (*n* = 23 for frequency of GP visit) and this missing data was reported in our previous paper [19]. Missing data was reported as is; hence this data was not excluded. The data was analyzed using SPSS version 21.

## Results

### Sample characteristics

Most participants (86.9%) were married (*n* = 351). The majority (77%) of participants were housewives (*n* = 311), 75.7% had a secondary level of education (*n* = 306) and more than half (60.3%, *n* = 244) were from below average annual income families (lower than €16,113). Descriptive statistics are presented in our previous paper [19].

### Mammography screening practices

**Fig. 1:**
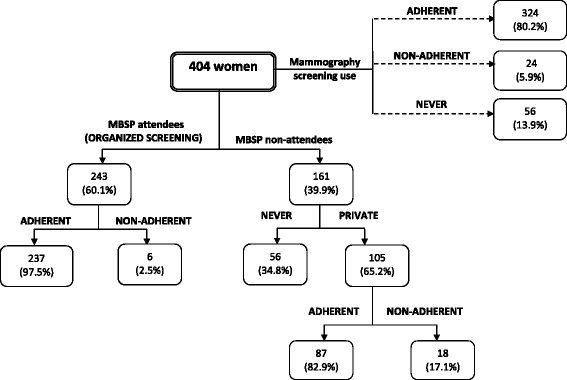
Mammography use in Malta

Mammography screening practices are presented in Fig. 1. Breast screening use (LIFETIME ATTENDEES) was reported by 86.1% of women (*n* = 348), of which 243 women underwent a mammogram at the MBSP. From those who did not undergo a mammogram at the MBSP (*n* = 161), 105 women underwent mammography elsewhere. No mammography was reported by 13.9% (*n* = 56) (LIFETIME NON-ATTENDEES).

### LIFETIME ATTENDEES versus LIFETIME NON-ATTENDEES subgroup analyses

Chi-square tests were performed to explore associations between lifetime attendees and non-attendees, and the following variables: sociodemographic factors, health status, knowledge, health beliefs and illness perceptions.

### Sociodemographic factors and health status

There was significant association between marital status and lifetime mammography (χ2 = 9.0, *p* = 0.030) such that a lower number of widowers attended for mammography (66.7%) when compared to women of other statuses (single, married, separated/divorced) (≥87%). The higher their family income, the more likely it is for a woman to undergo mammography in her lifetime (χ2 = 13.1, *p* = 0.011). In fact, all women who had a family annual income greater than €23,564 claimed that they acquired mammography during their lifetime while from those with a family annual income lower than €10,737, around one in every four women did not undergo mammography. In addition, those who do not drive are more likely not to attend for a mammogram (χ2 = 7.7, *p* = 0.006). Our data showed that 91.5% of drivers attended for a mammogram in their lifetime as compared to 81.9% of non-drivers. All women in our sample with a breast condition or disease attended for mammography in their lifetime when compared to 82.9% of women without a breast condition (χ2 = 14.2, *p* <  0.001). Moreover, those who had relatives or close friends with cancer were more likely to attend for mammography (χ2 = 8.3, *p* = 0.016).

No significant association was found between lifetime mammography and age (Independent samples t-test: *p* = 0.133), district (χ2 = 7.8, *p* = 0.802), owning a car (χ2 = 1.2, *p* = 0.267) or having an illness (χ2 = 0.1, *p* = 0.709). Although there was no significant association for level of education (χ2 = 5.4, *p* = 0.067) and occupation (χ2 = 5.7, *p* = 0.057), women with a higher education level and who were employed were more likely to undergo mammography in their lifetime (e.g. 93.2% {employed} versus 83.9% {housewives}). There was no significant association between having a family physician and lifetime mammography (χ2 = 3.5, *p* = 0.060). However, women who were not encouraged by their GP were more likely not to attend for a mammogram during their lifetime (χ2 = 4.9, *p* = 0.027).

### Knowledge of the recommended mammography frequency

Knowledge of mammography frequency was significantly associated with whether women had undergone a mammogram in their lifetime (χ2 = 28.5, *p* <  0.001). The main difference arises with those who said they were ‘unsure’ about the recommended mammography frequency (48% of the latter group did not undergo a mammogram in their lifetime), whereas for women who mentioned other mammography frequency options (i.e. ‘every year’; ‘every 1.5 years’; ‘every 2–3 years’), more than 86% of women from each individual latter groups had acquired a mammogram.

### Health beliefs

All sub-scale items for perceived barriers and cues to action for mammography use were found to be statistically significant (*p* <  0.05) (Table 1). Women tend to attend less for mammography if they are in agreement with or are undecided on the following: having a mammogram *‘would make you more anxious’, ‘more worried’, ‘more fearful about BC*’ and ‘*the procedure itself’*, is ‘*embarrassing’* and ‘*time-consuming’* and ‘*causes unnecessary radiation’*, have ‘*fear or distrust the medical team’*, ‘consider *other problems in life to be greater’* and feel they are ‘*not old enough to have a mammogram periodically’* (*p* <  0.001 respectively). Significant association is mirrored for the statement ‘*you fear having a mammogram because you know someone (family or friend) with breast cancer*’ (*p* <  0.001). When comparing pain and discomfort with mammography use, statistical significance is mirrored (*p* <  0.001), whereby the absolute majority of the undecided group (95.8%) do not attend for a mammogram in their lifetime whereas those who are in disagreement or in agreement (≥88%) attend for mammography.

**Table 1:** Health Belief items

	LIFETIME SCREENERS versus NON-SCREENERS	
Health Beliefs	χ2	*p*-value
There is no possibility of getting breast cancer *(r)*	8.4	0.077
Your chances of getting breast cancer are high	8.2	0.085
There may be the possibility of developing breast cancer in your lifetime	3.0	0.390
When you get a mammogram, you feel good about yourself	45.5	< 0.001*
When you get a mammogram, you do not worry as much about breast cancer	6.4	0.093
Having a mammogram will help you find lumps early in your breasts	19.1	< 0.001*
If you find a lump through a mammogram, the treatment for breast cancer may not be as bad	5.2	0.160
Having a mammogram will decrease your chances of dying from breast cancer	7.5	0.580
Having a mammogram will help you find a lump before it can be felt by yourself or a health professional	7.2	0.065
Having a routine mammogram would make you anxious about breast cancer	27.7	< 0.001*
Having a routine mammogram would make you worry	22.8	< 0.001*
You fear having a mammogram because you might find out that something is wrong	39.7	< 0.001*
You fear having a mammogram because you do not know the procedure or what to expect	145.8	< 0.001*
You fear having a mammogram because you know someone (family or friend) with breast cancer	20.0	< 0.001*
It is embarrassing for you to have a mammogram	40.4	< 0.001*
Undergoing mammography will be painful or uncomfortable	147.5	< 0.001*
Having a mammogram is time consuming	31.1	< 0.001*
You are discontent with Breast Screening personnel as they have been rude to you	n/a	n/a
You have fear or distrust in the medical team	32.9	< 0.001*
Having a mammogram would expose you to unnecessary radiation	27.9	< 0.001*
You have too many other problems in your life than to get a mammogram done	83.1	< 0.001*
You are not old enough to have a mammogram periodically	35.4	< 0.001*
If your GP advises you to attend for a mammogram, you will attend	54.4	< 0.001*
If your relatives or friends advise you to attend for a mammogram, you will attend	16.9	0.001*
If someone close to you has been diagnosed with breast cancer, you will attend for a mammogram	39.4	< 0.001*
Hearing about breast cancer and breast screening in the media or news makes you think about getting a mammogram	34.2	< 0.001*
Reminder letters would help you to get a mammogram	38.9	< 0.001*
Reminder phone calls or text messages would help you to get a mammogram	38.9	< 0.001*
Routine educational talks regarding breast cancer awareness would help you to get a mammogram	37.1	< 0.001*
You feel confident that if you had a mammogram done, any abnormalities in your breasts will be detected	0.6	0.960
You can arrange other things in your life to get a mammogram	49.2	< 0.001*
In case you need a mammogram, you will find a place to get it done	32.8	< 0.001*
You can make an appointment for a mammogram	36.0	< 0.001*
You can arrange transportation to get a mammogram	41.1	< 0.001*
You can talk to people at the breast screening centre about your concerns	n/a	n/a
You can find a way to pay for a mammogram if you need to	32.3	< 0.001*

*Statistically significant

*(r)* = *reverse scored*

^a^Chi-square test was applied for all health beliefs; hence the categorical answers were used to apply this test for association. For each question, respondents were asked to select a number between 1 and 5, where 1 = strongly disagree and 5 = strongly agree. For certain items, responses were re-grouped to ensure the feasibility of the Chi-square test

Those who underwent mammography tend to attend more for mammography if advised by their GP (χ2 = 54.4, *p* <  0.001) or by relatives or friends (χ2 = 16.9, *p* = 0.001). Those who are in disagreement that hearing about BC and BS in the media would trigger thoughts to get a mammogram tend to attend less. The absolute majority of those who are in disagreement that cues to action (such as ‘reminder letters’, ‘reminder phone calls’ or ‘text messages’) are effective, are more likely not to attend for mammography. There is also similar significant association for the vast majority of self-efficacy sub-scale items (*p* <  0.001) i.e. for attendees, the stronger is women’s confidence in arranging other things in their life to get a mammogram, while for the undecided group and those who are in disagreement with self-efficacy items are more likely not to attend for mammography screening.

### Illness perceptions

There is significant association for the emotional representation subscale items (*p* <  0.05) (Table 2). For lifetime non-attendees, the higher is their anxiety (χ2 = 8.3, *p* = 0.040) and fear (χ2 = 8.3, *p* = 0.039) of BC. The undecided group attend less for mammography when taking into account that their emotional state (χ2 = 12.9, *p* = 0.002) and their own behaviour (χ2 = 12.7, *p* = 0.002) is perceived to possibly cause BC. Those who agree that BC can be caused by their own behaviour (χ2 = 12.7, *p* = 0.002) or by a germ/virus (χ2 = 9.4, *p* = 0.009) attend less for mammography, while those who consider BC to have major consequences in life (χ2 = 9.9, *p* = 0.019) attend more.

**Table 2:** Illness Perception items

	LIFETIME SCREENERS versus NON-SCREENERS	
Illness Perceptions	χ2	*p*-value
The presence of a lump or thickening in the breast	1.8	0.611
Nipple discharge	2.3	0.509
Sudden nipple retraction	1.1	0.769
Change in shape or appearance of the nipple	1.2	0.743
Breast swelling, dimpling, redness or soreness of the skin	0.9	0.826
Skin changes of the breast	1.7	0.641
A sudden change in breast size	1.5	0.688
Aching breasts	1.5	0.820
Stress or worry	3.0	0.223
Your mental attitude (e.g. thinking about life negatively)	2.0	0.580
Family problems or worries	2.9	0.233
Overwork	7.9	0.052
Your emotional state (e.g. feeling down, lonely, anxious, empty)	12.9	0.002*
Your personality	3.0	0.391
Hereditary - it runs in the family	9.7	0.021*
Diet or eating habits	1.5	0.679
Poor medical care in the past	0.8	0.847
Your own behaviour	12.7	0.002*
Ageing	1.9	0.395
Smoking	1.8	0.601
Alcohol	1.2	0.538
A germ or virus	9.4	0.009*
Pollution in the environment	1.4	0.709
Altered immunity	2.5	0.469
Chance or bad luck	3.0	0.562
Accident or injury	3.6	0.460
Breast cancer will last a short time	5.8	0.120
Breast cancer is likely to be permanent rather than temporary	0.9	0.650
A patient with breast cancer goes through cycles in which her illness gets better and worse	5.8	0.215
Breast cancer has major consequences on a patient’s life	9.9	0.019*
Breast cancer will not have much effect on your life	6.1	0.189
Breast cancer would strongly affect the way others see you	7.8	0.100
Breast cancer has serious economic and financial consequences	5.0	0.174
Breast cancer would strongly affect the way you see yourself as a person	0.9	0.826
Breast cancer would threaten a relationship with your husband or partner	2.5	0.641
If you had breast cancer, your whole life would change	5.6	0.133
If you developed breast cancer, the chances of living a long life would decrease	4.9	0.179
There is a lot which you can do to control the symptoms if Breast Cancer occurs	0.7	0.948
The course of Breast Cancer will depend on your actions	2.9	0.400
Your actions will have an effect on the outcome of Breast Cancer	4.0	0.261
There is no treatment that will help to improve Breast Cancer	4.0	0.400
The treatment provided will be effective in controlling or curing Breast Cancer	3.1	0.371
The negative effects of Breast Cancer can be prevented or avoided by the treatment given	1.5	0.822
You have a clear picture and understanding of Breast Cancer	4.5	0.211
Breast Cancer is a mystery to you	2.1	0.720
You get anxious when you think about Breast Cancer	8.3	0.040*
Breast Cancer makes you feel afraid	8.3	0.039*
You get worried when you think about Breast Cancer	4.3	0.231

*Statistically significant

^a^Chi-square test was applied for all health beliefs; hence the categorical answers were used to apply this test for association. For each question, respondents were asked to select a number between 1 and 5, where 1 = strongly disagree and 5 = strongly agree. For certain items, responses were re-grouped to ensure the feasibility of the Chi-square test

### Health beliefs and illness perception constructs

The following 4 HBM and 1 CSM constructs were found to be significantly different when comparing lifetime mammography attenders and non-attenders: perceived benefits, perceived barriers, cues to action, self-efficacy (*p* <  0.001 respectively) and emotional representations (*p* = 0.033) (Table 3).

**Table 3:** Comparisons between mammography screening use and health beliefs/illness perception constructs. For all constructs, Mann Whitney test and Independent Samples t-test were applied to compare ‘LIFETIME ATTENDEES’ and ‘LIFETIME NON-ATTENDEES’

	LIFETIME ATTENDEES (*n* = 348)	LIFETIME NON-ATTENDEES (*n* = 56)	Test Statistic	*p*-value
Perceived Susceptibility	M = 9.6, SD = 1.0	M = 9.6, SD = 1.0	10,065.5^a^	0.669
Perceived Benefits	M = 24.0, SD = 1.8	M = 23.1, SD = 1.5	6816.5^a^	< 0.001*
Perceived Barriers	M = 27.5, SD = 4.9	M = 34.8, SD = 4.9	16,569.5^a^	< 0.001*
Cues to action	M = 27.3, SD = 3.2	M = 23.1, SD = 4.8	4306.0^a^	< 0.001*
Self-Efficacy	M = 24.8, SD = 2.7	M = 22.7, SD = 2.8	6114.5^a^	< 0.001*
Breast Cancer Identity	M = 30.6, SD = 2.3	M = 30.7, SD = 2.0	10,344.0^a^	0.434
Causes of Breast Cancer	M = 56.0, SD = 7.2	M = 57.4, SD = 6.9	-1.3^b^	0.186
Cancer Timeline: Acute/Chronic	M = 6.1, SD = 0.9	M = 6.2, SD = 0.9	10,213.5^a^	0.534
Cancer Timeline: Cyclical	M = 3.6, SD = 0.7	M = 3.4, SD = 0.7	8513.0^a^	0.069
Consequences	M = 28.2, SD = 2.5	M = 28.5, SD = 2.0	9909.0^a^	0.837
Personal Control	M = 11.8, SD = 0.8	M = 11.9, SD = 0.5	9890.0^a^	0.757
Treatment Control	M = 9.9, SD = 0.7	M = 10.0, SD = 0.5	10,592.0^a^	0.119
Illness Coherence	M = 7.0, SD = 1.1	M = 7.0, SD = 1.1	9857.5^a^	0.880
Emotional Representations	M = 12.2, SD = 2.1	M = 12.7, SD = 2.5	11,431.5^a^	0.033*

*Statistically significant, ^a^ Mann Whitney test, ^b^ Independent Samples t-test

The findings show that for women who acquire mammography during their lifetime, the higher is their agreement on perceived benefits to mammography uptake, while more cues to action and greater self-efficacy help women to undergo mammography. Higher perceived barriers to mammography screening and stronger emotional representations of BC are associated with no mammography use during a woman’s lifetime.

#### Predictors of mammography screening practices

We further explored which variables and constructs were most significant to women’s attendance (LIFETIME ATTENDEES versus LIFETIME NON-ATTENDEES). A number of logistic regression models were applied (Table 4) in order to examine the variables/constructs (independent variables) which are key to identifying differences between women who attended mammography during lifetime and non-attendees (dependent variables). Model 1 represents the demographics against attendance/non-attendance. Although ‘drive’ and ‘status’ variables were found to be significant (*p* <  0.05), this model was not found to provide any accuracy to predict non-attendance. Hence, demographics are not providing any useful prediction for the scope of this analysis. Model 2 focused on Health Belief variables only, which served as the independent variables for this model. This model predicted attendance with an accuracy of 98.3% and non-attendance with an accuracy of 48.2%. Five variables were found to be significant (*p* <  0.05) with an Odds Ratio (OR) that varies between 0.213 (fear of unknown procedure) and 3.327 (arrange transportation) for all the five variables. Model 3 focused on Illness Perception variables only, which served as the independent variables for this model. This model predicted attendance with an accuracy of 99.4% and non-attendance with an accuracy of 5.4%. Six variables were found to be significant (*p* <  0.05) with OR varying between 0.432 (fear of breast cancer) and 1.926 (major consequences in life). The above significant predictors from both models 2 and 3 were incorporated into a new single model (Model 4), both health beliefs and illness perception variables serving as the independent variables for this model. The model accuracy, when combining both scores, improved to 98.0% for attendance and 53.6% for non-attendance. The model retained six significant predictors (*p* <  0.05) with OR varying between 0.212 (fear of unknown procedure) and 3.202 (arrange transportation). When all individual Health Belief and Illness Perception items were incorporated into one model (Model 5), eight variables were found to be significantly different (*p* <  0.05) with OR varying between 0.149 (fear of unknown procedure) and 3.716 (arrange transportation). The accuracy of the model improved again to 97.1% for attendees and 58.9% accuracy for non-attendees. When the 14 constructs (not individual items) related to Health Beliefs and Illness Perceptions were used to construct a logistic regression model (Model 6), ‘perceived barriers’ (OR 0.776) and ‘cues to action’ (OR 1.196) were found to be the strongest and most significant predictors (p <  0.05) to describe the variance between the subgroups. However, the accuracy for predicting the non-attendees was found to be 37.5% and 96.6% for predicting attendance, which is inferior when compared to Model 4. No health status variables were found to be significant and were therefore not included in Table 4.

**Table 4:** Logistic Regression Models on lifetime mammography use (LIFETIME ATTENDEES versus LIFETIME NON-ATTENDEES) against different variables and different constructs

	B	SE	Wald	*P*-value	OR	95% CI	Model Accuracy Attendance	Model Accuracy Non-attendance
Model 1: Demographics							100%	0%
Drive	0.912	0.325	7.891	0.005	2.488	1.317, 4.700		
Status	0.591	0.224	6.987	0.008	1.807	1.165, 2.801		
Constant	−4.605	0.792	33.773	0.000	0.010			
Model 2: Health Beliefs							98.3%	48.2%
Fear of unknown procedure	−1.548	0.219	50.028	0.000	0.213	0.138, 0.327		
Other life problems	−1.213	0.302	16.130	0.000	0.297	0.165, 0.537		
Relative or close friend with breast cancer	0.383	0.187	4.218	0.040	1.467	1.018, 2.114		
Reminder letters	1.099	0.307	12.826	0.000	3.001	1.645, 5.475		
Arrange Transportation	1.202	0.410	8.605	0.003	3.327	1.490, 7.427		
Constant	−1.993	2.109	0.893	0.345	0.136			
Model 3: Illness Perceptions							99.4%	5.4%
Hereditary	0.579	0.233	6.179	0.013	1.784	1.130, 2.816		
Own behaviour	−0.554	0.213	6.774	0.009	0.575	0.379, 0.872		
Major consequences in life	0.655	0.255	6.627	0.010	1.926	1.169, 3.172		
Economic consequences	0.520	0.238	4.777	0.029	1.683	1.055, 2.683		
Threatens your relationship	−0.396	0.178	4.973	0.026	0.673	0.475, 0.953		
Fear of breast cancer	−0.840	0.280	9.038	0.003	0.432	0.250, 0.746		
Constant	1.060	1.828	0.337	0.562	2.888			
Model 4: Health Beliefs and Illness Perceptions							98.0%	53.6%
Fear of unknown procedure	−1.553	0.224	48.123	0.000	0.212	0.136, 0.328		
Other life problems	−1.239	0.310	15.973	0.000	0.290	0.158, 0.532		
Relative or close friend with breast cancer	0.407	0.189	4.618	0.032	1.502	1.036, 2.178		
Reminder letters	1.123	0.316	12.638	0.000	3.074	1.655, 5.710		
Arrange transportation	1.164	0.411	8.028	0.005	3.202	1.432, 7.163		
Own behaviour	−0.612	0.288	4.536	0.033	0.542	0.309, 0.952		
Constant	−0.240	2.306	0.011	0.917	0.787			
Model 5: Health Beliefs and Illness Perceptions							97.1%	58.9%
Poor medical care	0.878	0.360	5.970	0.015	2.407	1.190, 4.870		
Own behaviour	−1.195	0.380	9.893	0.002	0.303	0.144, 0.637		
Pollution	0.603	0.283	4.543	0.033	1.829	1.050, 3.185		
Possibility of developing breast cancer	−1.295	0.658	3.876	0.049	0.274	0.075, 0.994		
Fear of unknown procedure	−1.907	0.268	50.587	0.000	0.149	0.088, 0.251		
Other life problems	−1.478	0.331	19.976	0.000	0.228	0.119, 0.436		
Reminder letters	1.256	0.321	15.352	0.000	3.512	1.874, 6.584		
Arrange transportation	1.313	0.442	8.832	0.003	3.716	1.564, 8.831		
Constant	3.476	3.406	1.041	0.308	32.328			
Model 6: The 14 constructs							96.6%	37.5%
Perceived barriers	−0.253	0.039	43.157	0.000	0.776	0.720, 0.837		
Cues to action	0.179	0.041	19.169	0.000	1.196	1.104, 1.295		
Constant	5.192	1.688	9.460	0.002	179.859			

*B* unstandardized coefficients; *SE* standard error; *OR* odds ratio; *CI* confidence interval

## Discussion

The extant research identifies multifactorial reasons why women choose not to attend for mammography screening [9, 35–39], particularly psychological, socio-economic and practical factors [15, 28, 40, 41]. Hence, this study was carried out to provide an understanding of the determinants of lifetime mammography use among Maltese women who attend ‘anywhere’ and those who ‘never’ attend for mammography. This study found that four health belief constructs (perceived benefits, perceived barriers, cues to action, self-efficacy) and one illness perception construct (emotional representations) influence lifetime mammography screening practices among Maltese women. In particular, our findings show that women who perceive more barriers to mammography attendance (e.g. fear of pain, fear of the result), fewer benefits (e.g. lower belief in early detection), lower cues to action (e.g. no advice by a GP) and lower self-efficacy (e.g. lower confidence in one’s ability to arrange other things in life), and who have higher emotional representations of BC (e.g. greater fear, worry, anxiety and who consider other problems in life to be greater) were less likely to attend for mammography during their lifetime. This is consistent with Champion’s Health Belief Model and Leventhal’s Common-Sense Model of self-regulation. This also implies that women who have previously experienced mammography screening may already have established health-related behaviours [42] and have therefore already recognized the benefits of undergoing regular mammography use, have already overcome personal barriers to undergo mammography, have increased their self-confidence in getting screened throughout their lifetime and have higher levels of health motivation [23, 28, 42–44]. Therefore, efforts should be focused on identifying and encouraging attendance among women who have never participated in screening [44].

Our findings emphasize the importance of adapting interventions for women with lower socio-economic backgrounds, particularly since widowers, those having lower family incomes and non-drivers were found to be significantly associated with lifetime non-attendance in this study. These women are less likely to attend for screening anywhere. Women with socio-economic disadvantages in life are less likely to take part in any mammography screening. This relationship has been shown in previous literature [45]. Having a free-of-charge, invitational, organized screening programme is one of many interventions which would help to increase mammography use. This socioeconomic difference is re-emphasized in our previous study whereby household income has solely emerged as significantly associated with attendance to first invitation at the MBSP [19]. Although not statistically significant in this present study, women with a higher level of education and in employment were found to be more likely to attend than non-employed women and those with a lower education level. These socio-economic characteristics may serve as a proxy for interaction with other people, and in the degree of social integration during a woman’s lifetime. These findings may indirectly reflect social differences as well as the degree of equality regarding detection of BC and treatment received, and may help to identify prognostic factors amenable to intervention.

There were significant associations in this study between lifetime attendees and non-attendees regarding having a breast condition or BC in the family and the close relations, such that women with a breast condition or who had relatives or close friends with cancer were more likely to attend for mammography during their lifetime. Similarly, having a family member or close friends with BC was found to be associated with mammography attendance in other studies [46, 47] but contrast others [48–50]. Women most often play key roles as health managers and family caregivers [51–53] and this is not only reflected in that women more regularly than men are searching for health-related information on the Internet [54] but in women seeking a preventive action when faced with a prior personal or close relation experience that subsequently triggers them to engage in a health-related behaviour [50, 55, 56]. This corresponds with other research in other fields, particularly on mothers and children [57].

It has been acknowledged that lifetime non-attendees are an extremely difficult group to target and are a real challenge for screening management and public health officials [58]. For instance, structural and socio-economic factors such as age, income and marital status cannot be directly or easily modified [59]. Hence, although the exploration of such variables can help identify those at risk for a poor screening profile, such research offers little direction in terms of viable interventions. Therefore, in order to better understand which constructs are most significant to lifetime mammography non-attendees in Malta, our logistic regression analyses confirmed that *health beliefs* were the strongest and most important predictors to lifetime non-attendance and this result has been consistent across our previous research on first invitation to the MBSP [19], re-attendance [28] and adherence to timely mammography use [60]. This implies that lifetime non-attendees are women who were not motivated in health behaviour, have strong emotional representations of BS and BC, who highlight more barriers to screening, lower benefits and less cues to action because this is a new skill for them. This is evidenced by women who do not attend for mammography in other countries [59, 61] because they perceive greater barriers to BS.

Our data shows evidence that lifetime non-attendees were less encouraged by their GP to attend for a mammogram during their lifetime. However, it is also true that Maltese women tend to visit their GPs when they have a problem rather than on a routine basis [19]. While it is known that GPs are significantly more influential than relatives or friends at supporting the uptake of BS by mammography [55], women obtain information more often from friends and relatives than from official sources [62]. This reinforces the influence of word of mouth from friends and relatives as a means of screening promotion [55], supporting related promotional schemes worldwide [63–65]. However, while word of mouth is important, such initiatives are aimed at ensuring that information passed through word of mouth is based on factual information, rather than emotional reasons [55]. Although physician recommendation is critical for the provision of factual information (about BS, BC and adherence recommendations) [59, 66], many women still do not screen frequently enough [59]. Hence, it seems increasingly clear that interventions should be developed to target variables that are both amenable to change and for which there is scope for improvement, if breast screening rates are to be improved.

Emotional representations play a central role in models of both self-regulation and health behaviour [66] as well as in models regarding the “uptake” of health-promoting messages [67]. However, research cannot determine exactly what women are afraid of or how the diverse fear components are related to one another or to screening behaviour, particularly since contradictory findings across studies make it difficult to draw conclusions from the literature. Hence, fear, anxiety and worry are often termed to encompass nearly “everything” [59]. Our current study investigated barriers related to fear more specifically and we found that fear is certainly related to a breast cancer diagnosis, fear of pain/discomfort, fear of embarrassment, fear of the medical establishment, radiation, as well as general worry and anxiety. Similarly, other research found that fear is instilled due to an awaited result that may cause a negative impact on the self and on the family [59, 61, 68, 69], due to the pain perceived or experienced during the test [15, 44, 49, 70, 71], due to the sense of uncomfortableness whereby one exposes such an intimate body part in front of another person [44, 45, 71, 72], fear of the medical team [59, 73], fear that X-rays would cause more harm than good to the breast [15, 45] and nonspecific “cancer worry” [45, 49, 59, 74, 75] and general anxiety [76, 77]. Studies suggest that mammography-related anticipatory anxiety may contribute to poorer adherence [15, 69, 78] because women may avoid undergoing mammography to reduce their anxiety. It is possible that reports of mammography-related anxiety and catastrophizing thoughts related to mammography pain reflect women’s level of general anxiety [79, 80]. This may also operate as a barrier for relatives or friends to undergo mammography or attend a particular unit [81]. Hence, such concerns need also to be taken seriously to encourage long-term adherence among attendees by finding ways how to avoid pain and maintaining client satisfaction [82, 83]. Women can be prepared for mammography by informing them about possible short-lived pain or discomfort, preferably in the invitation letter or in screening campaigns [15]. Additionally, calming self-statements or distraction techniques could be utilised to reduce the fear of pain and embarrassment during the test [23].

The undecided group of women in this study tend to attend less for mammography screening, particularly those who are unsure about: (i) self-efficacy items such as whether they can arrange other things in life to get a mammogram, (ii) screening barriers such as whether mammography is painful or uncomfortable, (iii) illness perception items such as whether one’s emotional state or own behaviour causes BC, and (iv) mammography frequency recommendations). In all of our findings, limited knowledge was found to be significantly associated with attendance to the first screening invitation, re-attendance, lifetime mammography use and compliance with recommended time intervals. This calls for urgent renewed health education and tailored information on the importance of screening while addressing misunderstandings, debunking screening myths and improving knowledge gaps. All of our findings in this study, and when considered in the light of our previous results, can be used to lead the development of current non-existent, evidence-based interventions in Malta.

### Strengths and limitations

Our group of ‘real’ non-attendees came from the same target screening group, which further strengthens the value of our data. Additionally, the rich dataset allowed for diverse subgroup analyses, which facilitated an overview of lifetime screening practices, though not without possible response bias as a possible weakness. An additional strength is that the 121-item tool (MBSQ) contains information that makes it possible to adjust the analyses for potential confounders. Some aspects of study limitations should be considered. One limitation of the study is its cross-sectional design, which does not allow for the associations of non-attendance with socio-demographic factors such as age to be studied over time. Future research is needed to evaluate a potential cause effect relation. A problem in some of the analyses is the low number of ‘real’ non-attendees, hence a lower level of confidence in the results for this particular group. This may have led to a type I and/or type II error in relation to some of the analysed factors. Another limitation of this study is that self-reports for private mammography was used to measure lifetime mammography rather than objective data from private mammographic screening clinics. However, no national data records from private practices are currently available to date in Malta. Hence, self-reports for lifetime mammography use was the only possible method of data collection. The findings are likely to be generalizable and broadly applicable to other populations. Although limited to the Maltese population, the representation of our heterogeneous population derives from different parts of the country. However, given the potential for cultural differences, varied health care delivery systems, and socioeconomic factors between countries, the generalizability of study results may be somewhat limited.

## Conclusions

Our findings may be used to develop cognitive interventions aimed at enhancing perceived benefits, reducing perceived barriers, and modifying negative emotional representations to BC in order to motivate women to start undertaking mammography screening. In general, our results are in line with differences reported in the literature between screening attendees and non-attendees, such that non-attendees were less knowledgeable of the recommended mammography frequency, had attitudinal, emotional and motivational barriers, less socio-economic support and were less confident in themselves and the medical establishment. Additionally, our study showed that health beliefs were the most significant predictors to lifetime mammography screening behaviour. Hence, screening organizers and public health officials should target women’s perceived barriers and enhance cues to action when reaching out to non-attendees. Further qualitative research is required to clarify the determinants and consequences of emotional barriers, particularly fear among the ‘real’ non-attending cohort, and also to evaluate the need for a more targeted approach among this hardest-to-reach group in order to understand the complexity of their behavioural barriers.

## Abbreviations

B: Unstandardized coefficients
BC: Breast cancer
BS: Breast screening
CHBMS-MS: Champion’s health belief model scale for mammography screening
CI: Confidence interval
CSM: Common-sense model
GP: General practitioner
HBM: Health belief model
IPQ: Illness perception questionnaire
IPQ-R: Revised illness perception questionnaire
M: Mean score
MBSP: Maltese breast screening programme
MS: Mammography screening
OR: Odds ratio
SD: Standard deviation
SE: Standard error
